# Vasculogenic mimicry contributes to lymph node metastasis of laryngeal squamous cell carcinoma

**DOI:** 10.1186/1756-9966-29-60

**Published:** 2010-06-02

**Authors:** Wei Wang, Peng Lin, Chunrong Han, Wenjuan Cai, Xiulan Zhao, Baocun Sun

**Affiliations:** 1 Department of Pathology, Tianjin Cancer Hospital, Tianjin Medical University, Tianjin 300060, PR China; 2 Department of Pathology, Tianjin Medical University, Tianjin 300070, PR China; 3 Department of Otorhinolaryngology Head and Neck, Institute of Otorhinolaryngology, Tianjin First Central Hospital, Tianjin 300192, PR China; 4 Department of Pathology, Tianjin First Central Hospital, Tianjin 300192, PR China

## Abstract

**Background:**

Survival of laryngeal squamous cell carcinoma (LSCC) patients has remained unchanged over recent years due to its uncontrolled recurrence and local lymph node metastasis. Vasculogenic mimicry (VM) is an alternative type of blood supplement related to more aggressive tumor biology and increased tumor-related mortality. This study aimed to investigate the unique role of VM in the progression of LSCC.

**Methods:**

We reviewed clinical pathological data of 203 cases of LSCC both prospectively and retrospectively. VM and endothelium-dependent vessel (EDV) were detected by immunohistochemistry and double staining to compare their different clinical pathological significance in LSCC. Survival analyses were performed to assess their prognostic significance as well.

**Results:**

Both VM and EDV existed in LSCC type of blood supply. VM is related to pTNM stage, lymph node metastasis and pathology grade. In contrust, EDV related to location, pTNM stage, T stage and distant metastasis. Univariate analysis showed VM, pTNM stage, T classification, nodal status, histopathological grade, tumor size, and radiotherapy to be related to overall survival (OS). While, VM, location, tumor size and radiotherapy were found to relate to disease free survival (DFS). Multivariate analysis indicated that VM, but not EDV, was an adverse predictor for both OS and DFS.

**Conclusions:**

VM existed in LSCC. It contributed to the progression of LSCC by promoting lymph node metastasis. It is an independent predictors of a poor prognosis of LSCC.

## Background

Laryngeal squamous call carcinoma (LSCC) is the second main upper respiratory tract tumor behind lung cancer in incidence and mortality rates. Despite many advances in the diagnosis and treatment of the disease, its overall survival rate has remained unchanged (at approximately 35-70%) over the past several decades. It is mainly due to uncontrolled recurrence and local lymph node metastasis[1]. Thus, it is necessary to develope new therapeutic targets for LSCC that can take advantage of the unique qualities of this disease.

It is traditionally known that tumor invasion and metastasis mainly depend on angiogenesis. Histological examination of human tumor specimens has confirmed that increased vascularity is a common feature of LSCC. However, the results of studies associating microvessel density and various clinical pathological parameters and/or outcome are still inconclusive in LSCC[2]. In addition, clinical uses of anti-angiogenic agents for head and neck squamous cell carcinoma(HNSCC), including bevacizumab, sorafenib, sunitinib, are currently limited to small clinical trials, and several ongoing large-scaled trials up to this point. Single-agent anti-angiogenic drugs so far have not shown activity in unselected HNSCC patients, with a response rate of less than 4%[3,4].On the other hand, combinations of anti-angiogenic drugs with other treatments appear to be promising therapies, and biomarkers appear to have the potential to play an important role in anti-angiogenic treatment of LSCC in the future. Therefore, it is necessary to discover how blood supply contribute to LSCC biology, and to explore its characteristic biomarkers.

Vasculogenic mimicry(VM) is an alternative type of blood supplement formed by highly invasive and genetically dysregulated tumor cells with a pluripotent embryonic-like genotype[5]. Such tumor cells contributes to the plasticity and gain the ability to participate in the processes of neovascularization and ultimately constructing a fluid-conducting, matrix-rich meshwork[6]. Tumors exhibiting in VM related to more aggressive tumor biology and increased tumor-related mortality[5]. It has previously been described in many mesenchymal tumors such as melanoma[7], synovial sarcoma[8], rhabdomyosarcoma[8], and osteosarcoma[9], and now has spread to epithelial carcinoma, for example, inflammatory and ductal breast carcinoma [10], ovarian carcinoma[6,11], prostatic carcinoma [12]. We have previousely reported VM in synoviosarcoma, rhabdomyosarcoma and hepatocellular carcinoma [13,14]. However, no study exists to our best knowledge, examining whether VM effects in squamous cell carcinoma.

In the current study, we detected VM and the traditional endothelium-dependent vessel (EDV)in 203 cases of LSCC both prospectively and retrospectively, to compare their different significance on clinical pathology and prognosis. The results suggested LSCC with VM were predisposed to develop lymph node metastasis post operation. VM may be a predictor of lymph node metastasis for LSCC and poor prognosis instead of EDV. In addition, we expected that further exploration of specific biomarkers of VM will contribute to anti-angiogenesis therapy in LSCC.

## Materials and methods

### Patients and Tumor Samples

This study enlisted a total of 203 patients with histopathologically diagnosed LSCC treated at Department of Head and Neck Surgery of Tianjin Cancer Hospital's from January 1990 to January 2003. Data collection included patient gender, age at diagnosis, tobacco use, alcohol consumption, location, tumor size, pTNM stage, T classification, lymph node status, distant metastasis, recurrence, histopathological grade, radiology, and follow-up data. All of the LSCC patients considered in the study received the standard surgery protocol according to NCCN Clinical Practice Guidelines in Oncology Head and Neck Cancers (2008).All samples were taken by excision, bioptic specimens were excluded. Follow-up began from post-operation. The follow up was completed in January 2008. In the first year of follow-up, the patient had a routine visit every 2 months (six times a year). In the second year, the patient is seen every 3 months (four times a year); in the third year, every 4 months (three times a year); in the fourth and fifth years, twice a year. Thus all cases included in this study have been followed for at least 60 months except those patients who died before that time. The mean follow-up time was 80 months (range 2-219 months). Tumor size was defined as the maximum dimension of the resected neoplasm. The tumors were classified according to the TNM and AJCC/UICC systems (2002). The median age of the patients was 66 years (range, 32-77 years) at the time of diagnosis, representing that of the general population with laryngeal cancer. 40 of 203 patients (19.70%) received postoperative radiation therapy. Tianjin Cancer Hospital's ethics committee approved the study protocol.

### Immunohistochemistry

#### Main agents

Heat-induced epitope retrieval in citrate buffer (0.01 mol/L; pH 6.0) was applied to all slides before immunohistochemical staining. The primary antibodies against CD31 were purchased from Zhongshan Golden Bridge Biotechnology Co. Ltd., Beijing, PR China. The 0.5% periodic acid and Schiff solutions were made in the pathology department of Tianjin Cancer Hospital and confirmed to be effective in previous experiments.

#### Mono staining

Staining with primary antibodies against CD31 was performed on formalin-fixed, paraffin-embedded tissues with the SP-9000 kit (Zhongshan Golden Bridge Biotechnology Co. Ltd., Beijing, PR China).

#### Double Staining

First, CD31 immunohistochemical staining was applied; then the sections were treated with 0.5% periodic acid solution for ten minutes and rinsed with distilled water for two-three minutes. In a dark chamber, these sections were treated with Schiff solution for fifteen-thirty minutes. After distilled water rinsing, sections were counterstained with hematoxylin.

#### Evaluation of the Staining

VM was first identified with hematoxylin-eosin staining slides. It could be seen to be formed by tumor cells but not endothelial cells without hemorrhage, necrosis, or inflammatory cells infiltrating near these structures. CD31/periodic acid-Schiff (PAS) double-stained was then used to validate VM. It was identified by the detection of PAS-positive loops surrounding with tumor cells (not endothelial cells), with or without red blood cells in it. In CD31-stained slides, there were no positive cells in VM. Microvessel density (MVD) was determined by light microscopy examination of CD31-stained sections at the "hot spot". The fields of greatest neovascularization were identified by scanning tumor sections at low power (×100). The average vessel count of three fields (×400) with the greatest neovascularization was regarded as the MVD. The MVD was classified as either high (≥17.53) or low (<17.53); 17.53 was the median value of MVD.

### Statistical Analysis

Analyses were conducted in the SPSS software version 11.0 (SPSS, Inc., Chicago, IL). The Kruskal-Wallis Test was used to compare the positive rate of VM with clinical pathologic variables, as appropriate, while using One-Way ANOVA to analyze the relationship with clinical pathologic data. Overall and disease-free survival curves were plotted using the Kaplan-Meier method and different subgroups were compared using the log-rank test. Patients who dropped out during follow-up or died due to diseases other than laryngeal cancer were treated as censored cases. The Cox regression model was used to adjust for potential confounders. Comparison MVD expression between VM-positive and VM-negative group used *t *test. Significant level was set at 0.05. P values are two-tailed.

## Results

### Evidence of VM and EDV in LSCC

**Figure 1:**
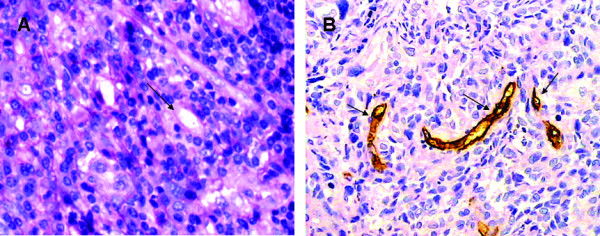
**Identifying VM and EDV in human sample of LSCC by CD31and PAS double staining**. A.) The VM channel (black arrow) in human sample is formed by laryngeal cancer cells. There are red blood cells in the center of the channel. PAS-positive substances line the channel and form a basement membrane-like structure (pink). Note the absence of necrosis and hemorrhage in the tumor tissue near the VM channel (original magnification: ×400). B.) Endothelium-dependent vessels (black arrows) are lined by spindle-shape endothelial cells, which are stained by CD31 (brown). The vessels' basement membrane is positive for PAS staining (pink) (original magnification: ×400).

Both VM and EDV existed in LSCC. Forty-four (21.67%) of 203 cases were VM-positive by double-staining. VM appeared to be PAS-positive loops surrounding tumor cells (not endothelial cells), with or without red blood cells. In CD31-stained slides, there were no positive cells in VM (Fig. 1A). While endothelium dependent vessel showed a CD31-positive endothelial cell to form the vessel wall (Fig. 1B).

### Characteristics and follow up of patients

Among the 203 patients, there were 154 men (75.86%) and 49 women (24.14%). The mean age at diagnosis was 66 years, ranging from 32 to 77 years. 166 (81.77%) cases reported history of tobacco use, and 37 (18.23%) cases without. 91 (44.83%) cases indicated history of alcohol consumption and 112 (55.17%) cases without. Patients with tumors located at super glottic were 93 (45.81%) cases, at glottic were 93 (45.81%) cases, and at subglottic were 17 (8.37%) cases. Patients in pTNM stage I, II, III and IV were 25 (12.32%), 60 (29.56%), 62 (30.54%) and 56 (27.59%), respectively. Patients in different T classification T1, T2, T3 and T4 were 27 (13.30%), 93(45.81%), 44(21.67%) and 39(19.21%), respectively.151(74.38%) patients showed lymph node metastasis at diagnosis, and 19 (9.36%) patients appeared to show distant metastasis postoperative. In addition, histological grade 1 was in 30 (14.78%), grade 2 was in 149 (73.40%) and grade 3 was in 24 (11.82%) cases.

The mean follow-up time was 80 months (range 2-219 months). 121 patients (59.61%) were alive when the follow up ended. Eighty-two patients (40.39%) died as a result of their malignancy. The median DFS was 56 months. Local recurrence and local lymph node metastasis was observed in 157 patients (77.34%). The mean period from initial surgery to the first local recurrence or metastasis was 63.71 months (range 1-213 months). Nineteen (9.36%) patients developed distant metastasis. The metastatic sites included lung (*n *= 9), bone (*n *= 4), liver (*n *= 3), mediastinum (*n *= 2), and multiple concomitant metastasis (*n *= 1, including thoracic vertebrae, spinal cord and tibia).

### Clinical significance of VM in LSCC patients compared with EDV

Clinical significance of VM and EDV are listed in Table 1. The positive rate of VM was significantly higher in progressive stage (III and IV) than primary stage (I and II) (27.97% vs. 12.94%) (*p *= 0.010) clinically, and it was significantly greater in patients with local lymph node metastases than those without local lymph node metastasis (36.53% vs. 16.56%) (*p *= 0.003). In addition, the positive rate of VM became higher with the raise of histopathological grade: grade 1(6.67%), grade 2 (20.13%), grade 3 (50.00%) (*p *< 0.0001). And the incidence of VM did not differ with respect to the patients' gender, age, tumor size, T stage, tumor location, recurrence or distant metastasis (all *P *> 0.05).

**Table 1:** Comparing clinicalpathologic significance of VM and EDV

factor		VM			MVD		
	+	-	**χ^2^**	*P*	( ± S)	F/t*	*P*
Gender			0.881	0.380		1.228*	0.269
M	34	118			17.8739 ± 6.82709		
F	10	42			16.6340 ± 6.08995		
Age			0.370	0.712		0.108*	0.742
≥60	22	85			17.4393 ± 6.92216		
<60	22	74			17.7514 ± 6.57988		
							
Tobacco use							
Yes	37	129	0.202	0.653	17.3863 ± 6.67757	0.808*	0.370
No	7	30			18.4865 ± 6.97671		
Alcohol consumption			0.608	0.436		0.008*	0.927
Yes	22	69			17.5388 ± 6.43099		
No	22	90			17.6259 ± 6.99013		
Location			2.213	0.331		3.550	0.031
Super glottic	24	69			18.2441 ± 7.14615		
glottic	18	75			16.3786 ± 5.94319		
subglottic	2	15			20.3667 ± 7.35727		
pTNM			6.570	0.010		7.419*	0.007
I+II	11	74			16.0306 ± 6.19107		
III+IV	33	85			18.5977 ± 6.91980		
Tumor size (cm)			0.220	0.639		0.974*	0.325
≥3	20	66			18.1306 ± 6.22807		
>3	24	93			17.1872 ± 7.07416		
T stage			1.278	0.734		3.396	0.019
T1	6	21			13.8593 ± 5.61853		
T2	17	76			17.7731 ± 6.43417		
T3	11	33			17.9143 ± 6.69789		
T4	10	29			18.8667 ± 7.50099		
Nodal status			9.097	0.003		0.019*	0.892
N-positive (N1, N2, N3)	19	33			17.4769 ± 6.50208		
N-negative(N0)	25	126			17.6247 ± 6.82606		
Distant metastasis			1.535	0.215		4.077*	0.045
Yes	2	17			20.4684 ± 6.86740		
No	42	142			17.2186 ± 6.65992		
Recurrence			0.005	0.994		0.679*	0.498
Yes	7	26			18.3152 ± 6.59413		
No	37	133			17.4455 ± 6.76481		
Histopathological grade			15.531	0.000		0.209	0.811
1	2	28			16.8967 ± 5.69443		
2	30	119			17.7532 ± 7.12289		
3	12	12			17.4167 ± 5.42896		

VM: vasculogenic mimicry; EDV: endothelial dependent vessel; LSCC: laryngeal squamous cell carcinoma; TNM: tumor, node, metastasis.

We performed immunohistochemical staining for CD31, a classic endothelial cell marker, to label endothelial dependent vessel, and analyzed whether it was associated with tumor clinicopathologic characteristic. The results showed MVD was correlated to location (*p *= 0.031), pTNM stage (*p *= 0.007), T stage (*p *= 0.019) and distant metastasis (*p *= 0.045). While, showed no association between MVD and gender, age, tobacco use, alcohol consumption, tumor size, lymph node metastasis, recurrence or histopathological grade (all *P *> 0.05).

### Survival analysis

**Figure 2:**
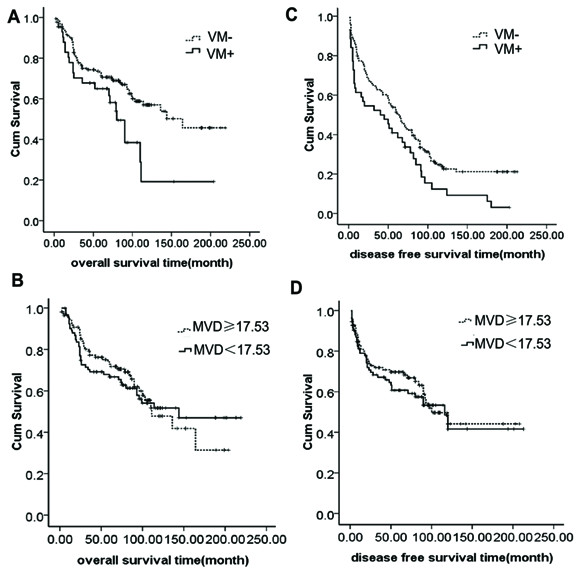
**The curves of overall survival and disease-free survival according to VM and MVD in 203 patients with LSCC**. A.) Overall survival according to VM positive and VM negative (*p *= 0.014). B.) Overall survival according to high MVD (MVD≥17.53) and low MVD (MVD＜17.53) (*p *= 0.772). 17.53 was the average MVD of 203 cases of LSCC patients. C.) Disease-free survival according to VM positive and VM negative (*p *= 0.011). D.) Disease-free survival according to high MVD and low MVD (*p *= 0.847).

Univariate analysis showed that survival of VM-positive patients was significantly poorer than that of VM-negative patients in OS (*p *= 0.014) (Fig. 2A). Furthermore, pTNM stage (*P *= 0.009), T classification (*P *= 0.013), nodal status (*P *= 0.013), and histopathological grade (*P *= 0.038), tumor size (*P *= 0.028), radiotherapy (*P *< 0.0001) correlated with OS. However, there was no significant association between OS and gender, age at diagnosis, tobacco use, alcohol consumption, location, distant metastasis, recurrence and MVD (Fig. 2B) (all *P > *0.05; Table 2). Multivariate analysis indicated that the presence of VM (risk ratio (RR) = -2.117, *P *= 0.003), recurrence (RR = -1.821, *P *= 0.020) and pTNM stage (RR = 1.367, *P *= 0.009) were adverse predictors for OS (Table 3), while radiotherapy were indicators of a good prognosis of OS (RR = 2.872, *P *< 0.0001).

**Table 2:** Univariate analyses of factors associated with recurrence, metastasis and survival

Variable	Overall Survival		Disease-Free Survival	
	χ^2^	*P*	χ^2^	*P*
Sex, male vs female	1.809	0.179	0.690	0.496
Age, y, ≥60 vs <60	0.075	0.784	0.342	0.559
Tobacco, Yes vs No	2.371	0.124	2.661	0.103
Drink, Yes vs No	0.013	0.911	0.648	0.421
Location, Super glottic vs glottic vs subglottic	0.585	0.746	6.035	0.049
pTNM stage, Ivs II vs III vs IV	11.600	0.009	4.592	0.204
T classification, T1 vs T2 vs T3 vs T4	10.744	0.013	6.915	0.075
Nodal status, N-positive vs N-negative	6.238	0.013	0.583	0.445
Distant Metastasis, Yes vs No	0.042	0.837	0.374	0.541
Recurrence, Yes vs No	12.386	<0.0001	0.043	0.836
Histopathological grade, 1 vs 2 vs 3	6.529	0.038	1.274	0.529
Tumor size, cm, ≥3 vs <3	4.809	0.028	10.364	0.001
Surgery modality (cervical neck dissection) Yes vs No	0.672	0.412	1.122	0.290
Radiotherapy, Yes vs No	26.752	<0.0001	27.750	<0.0001
MVD, <17.53 vs ≥17.53	0.084	0.772	0.037	0.847
VM, Yes vs No	6.054	0.014	6.535	0.011

VM: vasculogenic mimicry; MVD: micro vessel density.

**Table 3:** Multivariate analyses of factors associated with recurrence, metastasis and survival

	Variable	Hazard Ratio	95% Confidence Intervals		*p*
			
			lower	upper	
Overall Survival	VM, Positive vs Negative	-2.117	1.286	3.425	0.003
	Recurrence, Yes vs No	-1.821	1.363	3.639	0.020
	TNM stage, Ivs IIvs IIIvs IV	1.367	1.080	1.732	0.009
	Radiotherapy, Yes vs No	2.872	1.764	4.678	<0.0001
					
Disease-free Survival	VM, Positive vs Negative	-1.733	1.202	2.498	0.003
	Radiotherapy, Yes vs No	2.756	1.893	4.012	<0.0001

VM: vasculogenic mimicry; MVD: micro vessel density.

In addition, univariate analysis of DFS showed that VM (*P *= 0.011) (Fig. 2C), location (*P *= 0.049), tumor size (*P *= 10.364) and radiotherapy (*P <*0.0001) were proposed to correlate with DFS. While, gender, age at diagnosis, tobacco use, alcohol consumption, pTNM stage, T classification, nodal status, distant metastasis, recurrence, histopathological grade and MVD (Fig. 2D) (all *P > *0.05; Table 2) showed no correlation with DFS. Multivariate analysis showed that VM (RR = -1.733, *P *= 0.003) and radiotherapy (RR = 2.756, *P *< 0.0001) were independent prognostic factors for DFS (Table 3).

### Relationship between VM and EDV

To elucidate on the relationship between VM and EDV, the MVD between the VM-positive group and VM-negative group was compared. This determined patients of VM-negative group had a higher MVD (18.3403 ± 6.92318) than the VM-positive group (14.8643 ± 5.18685) (t = 3.096, *p *= 0.002) (Table 4). Correlation analysis revealed a negative correlation between VM and MVD (r = -0.198, *p *= 0.005).

**Table 4:** Correlation between VM and MVD of 203 LSCC patients

	n	**MVD **(** ± S)**	*t*	*P*
VM+	44	14.8643 ± 5.18685	3.096	0.002
VM-	159	18.3403 ± 6.92318		

VM: vasculogenic mimicry; MVD: micro vessel density.

## Discussion

This study confirmed VM as a new type of blood supply in LSCC by double staining. Angiogenesis (the formation or sprouting of endothelium-lined vessels from pre-existing vessels) and vasculogenesis (the difference between precursor cells and endothelial cells which develop *de novo *vascular networks) are two kinds of traditional blood types [15]. Both have been reported in LSCC[16]. VM is a new pattern of matrix-rich networks surrounding tumors cells, being reported firstly in melanoma by Maniotis in 1999 [5]. It refers to the *de novo *generation of tumor microcirculation without participation by endothelial cells; it is independent of angiogenesis. Furthermore, it is not a vasculogenic event for the true vasculogenesis results in endothelial cell-lined vessels' *de novo *formation. Majority of research on VM focuses on mesenchymal tumor [8,9,17], while only a few delve into epithelial tumor [6,10,11,18]. To date, there is dearth of research discussing squamous cell carcinoma. Thus, this study identifies VM existence in LSCC, in attempt to explain why anti-angio/vaculogenesis treatment remains to be clinically ineffective.

There is still no affirmative conclusion on the prognostic significance of the endothelium marker among CD31, CD34 and CD105. A long-term prognostic significance of angiogenesis in breast carcinomas compare with Tie-2/Tek, CD105, and CD31 immunocytochemical expression showed both CD31 and CD105 correlated with poorer survival [19]. Menio et al study on lung cancer reported that CD34-MVD and tumor vessel invasion not CD105, correlate with poor survival on multivariate analysis[20]. We selected CD31 to label endothelial-dependent vessel for the reasons: Because CD31/CD34 is a pan endothelial marker, and hence stains nearly all blood vessels, both stable vessels trapped inside the tumor and neoangiogenesis. However, CD105 (endoglin) is a proliferation-associated and hypoxia-inducible protein abundantly expressed in angiogenic endothelial cells. It is demonstrated that antibodies against CD105 reacted preferentially with active endothelial cells of angiogenic tissues. CD105 is a marker of neoangiogenesis and only stains a smaller proportion of blood vessels[21]. On the other hand, VM is an alternative type of blood supplement different from endothelium-lined vasculature. It is becoming evident that VM, the intratumoral, tumor-cell-lined, ECM-rich, patterned network, can provide an extra vascular fluid pathway, now known as the fluid-conducting meshwork[22,23]. Here, we compared clinical significance of VM with CD31-MVD, to disclose their different contribution to tumor biology. Thus, we choose CD31 to label endothelial-dependent vessel rather than CD105 was in order to reflect the whole blood supply in a tumor, for both newly-forming vessels and stable vessels trapped inside the tumor acted in tumor invasion and metastasis. More work should be done in the future to enrich the theory of tumor blood supply pattern, which may provide reasonable theoretic evidence for tumor anti-angiogenesis.

In the current study, we identified that the positive rate of VM in LSCC is 21.67%, which is different from other tumors, such as inflammatory and ductal breast carcinoma (7.9%), ovarian carcinoma(36.4%), melanoma(5.3%), rhabdomyosarcoma(18.8%), and synovial sarcoma(13.6%). That is probably due to different tissue origin and judgment criteria variable across labs. More investigation of a larger sample is needed to illustrate the mechanism of VM formation in different tissue.

Previous research has demonstrated VM existed in most tumors, being a functional microcirculation [24,25], correlated with poor clinical outcomes among tumor patients [14,26]. The majority of studies in vitro have focused on the mechanism, until recently. However, relatively few studies have interpreted VM's influence on a tumor's overall biological behavior using a large sample. In addition, there still no data which describes a significant difference between VM and other patterns of blood supply. In this study, we compared the significance of clinicopathology and prognosis between VM and EDV. This retrospective study of 203 LSCC patients showed that VM is associated with lymph node metastasis, pTNM stage and histopathology grade in LSCC. While EDV correlated with tumor location, pTNM stage, T stage and distant metastasis. This indicated that both VM and EDV played an important role in tumor progression.

Our study showed that VM is related to local lymph node metastasis intimately, which is an important feature and a key prognostic factor of LSCC[27]. It is different from a previous study[28], which reported that patients with breast carcinomas engaged in VM and had a higher rate of distant metastasis (liver, lung, and bone), but failed to find a significant correlation with lymph node metastasis status. In our study of 203 LSCC, only 9.36% appeared to have distant metastasis, while 74.38% developed local lymph node metastasis. We deduced from this that VM in LSCC may own the specific ability to facilitate metastasis by some modality. More studies are warranted to elucidate the effects of VM which use a larger sample on local lymph node metastasis in different types of tumors.

VM in tumors plays an important role in tumor aggression [5]. We also found VM is more common in the advanced stage of LSCC than in the primary stage. However, these results are different than the observations from a breast cancer study by Shirakawa et al[28], which showed that the VM group did not exhibit a more advanced pTNM stage than the non-VM group. However, there was no difference of VM exhibition among different T stage founded in Shirakawa's and our studies. We suggested that the discrepancy result may due to different influence of VM on local lymph node metastasis or distant metastasis in diversity tumors. Therefore, the impact of VM on the survival of patients with LSCC needs to be confirmed further by some international collaboration of studies and systematic reviews by meta-analysis.

In addition, we founded that positive rate of VM increased with the increase of histopathology grade, which is consistent with a previous study of hepatocellular carcinoma [14]. Nasu et al's [29]*in vitro *study demonstrated that VM was linked to the aggressive tumor cell phenotype. Another *in vitro *study [6] also found that high invasive melanoma cell line MUM-2B, expressing both epithelial and mesenchymal phenotype was able to form VM, while MUM-2C, a low invasive melanoma cell line expressing only mesenchymal phenotype, failed to form VM. Taken together, these studies imply that the lower histopathology grade of LSCC owning more cell heteromorphism, can change cancer plasticity by genetic reversion to a pluripotent embryonic-like genotype to ultimately form VM. However, in the study of EDV, it was both VM and EDV were related to pTNM, while no association was found between EDV and pTNM rather than distant metastasis. Therefore, we speculated that both VM and EDV contributed to LSCC progression, but through a diverse pathway. VM is a distinct pattern of blood supply from EDV. In general, VM may facilitate invasion and local metastasis in LSCC, indicating its role on aggressive behavior.

Previous study demonstrated that tumors with VM exhibited poor survival[9,13]. We found that VM was an unfavorable prognostic factor of LSCC patients both in OS and DFS, whereas EDV was not an independent predictor of outcome, consistent with Sun et al's [14] investigation in hepatocellular carcinoma. Traditional microvessel density counts [30,31] within vascular hot spots of tumors using endothelial markers reflect only the vascular status of endothelial dependent vessel in a tumor, but ignore other patterns of the vascularity, including VM, leading to low microvessel density in the different tumor types. However, Eberhard et al[32] demonstrated that endothelial dependent vessel alone, there is wide variance in the endothelial proliferation index among the various tumor types. This indicated that there is marked heterogeneity of vasculature in human tumors. It is necessary for us to account for all types of blood supply and their contribution to tumor behavior when evaluating its clinical and prognostic value. Moreover, the phenomenon of VM existence can partly explain why we failed in anti-angiogenesis treatment of LSCC.

How do VM and EDV play their individual role in one neoplasm during tumor growth? In our retrospective of 203 cases LSCC, presentation of VM showed a negative correlation with EDV. Further investigation in vivo needs to be performed in order to detect the presence of VM and EDV to disclose the relationship between VM and EDV in the same tumor in a time-dependent way.

## Conclusions

In conclusions, our results suggest that VM might be a new target of anti- vasculogenesis/angiogenesis therapy for LSCC. Those who rely on conventional markers of tumor "vascularity" as prognostic markers, and who are developing anti-cancer therapies by targeting angiogenesis should exercise caution concerning VM when interpreting their results. Vasculogenic mimicry is one example of the remarkable plasticity demonstrated by aggressive melanoma cells and suggests that these cells have acquired an embryonic-like phenotype. Several factors are involved in VM formation, including microenvironment, interaction between tumor cells and surrounding tissue, tumor cells changing to endothelial genotype by expressing embryo genotype. Further studies are needed to elucidate the specific molecular mechanism of VM in LSCC on order to explore new therapies target, and to contribute to anti-vasculogenesis/angiogenesis therapy for vasculogenic mimicry in LSCC.

## Competing interests

The authors declare that they have no competing interests.

## Authors' contributions

Before submission, all authors read and approved the final manuscript. Among the authors, WW designed the study, performed all experiments, and drafted the manuscript. While ZXL and LP collected the materials and conducted the statistical analysis. HCR participated in the instruction of the experiment, while CWJ revised the manuscript critically to ensure important intellectual content. WW and LP read and reviewed the sections, and performed follow-up observations on all patients. SBC provided the study concept and participated in its design and coordination.
